# Effect of superheated steam roasting with hot smoking treatment on improving physicochemical properties of the adductor muscle of pen shell (*Atrina pectinate*)

**DOI:** 10.1002/fsn3.674

**Published:** 2018-05-24

**Authors:** Md. Mohibbullah, Na Eun Won, Jong‐Hoe Jeon, Jeong Hyeon An, Yeseul Park, Hari Kim, Khawaja Muhammad Imran Bashir, Sun‐Mee Park, Young Soo Kim, Sung‐Joon Yoon, Jae Hak Sohn, Andre Kim, Jae‐Suk Choi

**Affiliations:** ^1^ Seafood Research Center IACF Silla University Seo‐gu Busan Korea; ^2^ Research Center for Extremophiles and Microbiology College of Medical and Life Sciences Silla University Sasang‐gu Busan Korea; ^3^ Major in Food Biotechnology Division of Bioindustry College of Medical and Life Sciences Silla University Sasang‐gu Busan Korea; ^4^ EBADA Fishery Co. Ltd. Seo‐gu Busan Korea; ^5^ Major in Pharmaceutical Engineering Division of Bioindustry College of Medical and Life Sciences Silla University Sasang‐gu Busan Korea

**Keywords:** adductor muscle, *Atrina pectinate*, hot smoke, physicochemical properties, superheated steam

## Abstract

The adductor muscle of the pen shell *Atrina pectinata* (AMPS) is a popular protein‐enriched food item in Asian Pacific countries, and has only been marketed in the frozen condition, as a result browning and decreased sensory attributes occur. To overcome these problems, superheated steam roasting (at 270°C for 4 min) combined with the hot smoke (10 min) using a selective Oak sawdust was employed to develop a new AMPS product yielding high physicochemical properties during storage periods (0–13 days) especially at 10°C. The processed AMPS showed high sensory preferences because of good odor, color, and textural properties. It also significantly inhibited bacterial growth, volatile basic nitrogen, thiobarbituric acid‐reactive species, and pH changes, and eventually possessed higher nutritional composition with low trimethylamine N‐oxide level. Results indicate that saturated steam allows AMPS at good physicochemical conditions, whereas hot smoke‐derived aroma compounds prolong its shelf life through antioxidant and antimicrobial activities.

## 1 INTRODUCTION

The pen shell *Atrina pectinate* is a bivalve with large wedge‐shaped structure, belonging to the family Pinnidae (An, Lee, & Dong, 2012). In Korea, *A. pectinate* is known as Khi‐Jo‐Gae and one of the popular and commercially important seafood species, because of its high nutritional value and acceptance to the consumer levels as well. *A. pectinate* is found mainly in muddy to sandy substrates either with patches in existence or the formation of small clusters. The geographic locations of this benthic species are distributed in the areas of Indo‐western pacific region, southeastern part of Africa to Malaysia and New Zealand, Japan, and Korea (FAO 1998; Rosewater, 1961). A large number of Asian Pacific countries have shown their great interest in the promotion of culture and production of pen shell species, and marketed its edible part; especially adductor muscle and mantle, which are popular items for their tastes (FAO 1998; Lee et al., 2006). In Korea, the number of pen shell farms was dramatically increased with respect to consumer interest, of which annual production of *A. pectinate* was reported to be approximately 3,743,071 tons (7,983,103,620 $) in 2017 (Statistics Korea 2017).

Currently, the adductor muscle from *A. pectinate* is commonly found in the market in frozen storage condition to maintain quality and functionality. Chen, Wu, and Pan (2015) reported that browning occurred during freezing conditions as a result of decreased sensory quality and market value of the pen shell items. The uses of chemical food additives or attractants in the extension of shelf life has been widely studied (Bashir, Kim, An, Sohn, & Choi, 2017). Moreover, chitosan combined with glutathione was found to have preserving effects on adductor muscle of the pen shell while keeping at frozen conditions (Chen et al., 2015). The coating substances from natural products containing bioactive ingredients have increasingly shown as non‐toxic and safety materials for preserving foods. (Bashir et al., 2017; Chen et al., 2015). Since adductor muscle possesses high nutrients than those of the other parts of the pen shell, it can easily be deteriorated during storage conditions. Keeping in mind, to develop a food product, the present efforts are being observed in combination of the treatment of superheated stream with hot smoking using natural aroma‐enriched sawdust in order to extend the shelf life of adductor muscle of the pen shell.

One of the traditional and widely used methods for processing and preservation of aquatic food products is smoking, where an incomplete burning smoke released from wood is readily transferred to the surface of the smoked goods (Oğuzhan Yildiz, 2015). This technique is mainly used for long‐term preservation of the fish products. In the present study, high temperature (>60°C) was used to smoke the adductor muscle of the pen shell using a high‐voltage electrostatic field. Smoked products have a unique color and flavor, which might be due to the presence of various complex compounds derived from wood or sawdust, including phenols, ethers, esters, hydrocarbons, acids, alcohols, ketones etc. (Bashir et al., 2017; Goulas & Kontominas, 2005; Guillén & Errecalde, 2002). However, shelf life of smoked products is greatly influenced by the initial bacterial contamination, decrease in water activity because of brining and predrying method, existence of smoke substances, and oxygen, humidity, and temperature conditions during storage. Therefore, we used superheated steam process to dry samples with transparent and colorless hot‐dry gas, before exposure to smoking. Bórquez, Canales, and Quezada (2008) reported that superheated steam in press‐cake of mackerel fish resulted in a relatively low moisture content with high levels of valuable fatty acids. Thus, it is possible to keep the inherent flavor, color, and texture of the food products when combined with hot smoking technique to add natural aroma compounds in the smoke of sawdust having a shelf life prolonging ability. Additionally, it has been found that superheated steam can synergistically retard the oxidation of vitamin C and browning of food and eventually prolong the shelf life of food with relatively greater freshness (Choi et al., 2013).

The pen shell *A. pectinata* has been reported to have various biofunctional effects such as anticancer activities (Park, Shin, Lee, & Bae, 2005), and antibacterial effect of its isolated peptide (Yoo et al., 2011) as well as a rich source of nutritional elements like protein, lipid, glycogen, and polysaccharide (Baik, Kim, Chung, Choo, & Park, 2001; Cao et al., 2015; Yurimoto, Yoshida, & Maeno, 2008). Preserving the nutrients, at best, in the fisheries product is one of the major challenges in the food industry. Till now, no reports are available on superheated steam technology in combination with hot smoking treatment of the adductor muscle of pen shell A. pectinate. The present study offers high‐ quality food product especially for the adductor muscle of pen shell, with the purpose of extending their shelf life during storage condition.

## 2 MATERIALS AND METHODS

### 2.1 Collection and preparation of *A. pectinata*

The pen shell *A. pectinata* was harvested along the southern coast of the Korean peninsula in the year of 2017 and obtained almost the same size and weight. The adductor muscles from *A. pectinata* were sectioned in a dimension of 2 × 5 × 1.4 cm and average weight of 12.5 ± 6 g, and divided into three groups, namely (i) raw sample without treatment, (ii) superheated steam roasting sample, and (iii) superheated steam roasting with the hot smoke sample.

### 2.2 Roasting of adductor muscles by superheated steam

The sample was used for superheated steam roasting in the Aero Steam Oven (DFC‐560A‐2R/L, Naomoto Corporation, Osaka, Japan) at 270°C for 4 min roasting (Optimized with the basis of color, odor, flavor, and overall acceptance criteria; data not shown). Prior to hot gas drying, a time‐controlled electric smoker (Braai Smoker; BSTD6, Bradley, Canada) generated steam until it reached the superheated state. During this time, superheated steam was moved into the drying chamber and passed through a tray (42 × 47 × 4 cm). Superheated steam can be recirculated using a centrifugal fan. The roasted samples were vacuum‐packed in polyamide/low‐density polyethylene bags using LOVERO^®^ vacuum sealer (Sambo Tech. Corporation, Gyeonggi‐do, Korea) and then stored at 4°C until use.

### 2.3 Brine salting and smoking

Superheated steamed fillets were immersed in brine solution containing 8% NaCl at a ratio of 1:1 (w/w) for 24 hr. Then each sample was kept at 30°C for 30 min to drain excess water. After that, the sample was transferred to the smoke chamber for smoking generated by the combustion of sawdust from the woods, smoked at 75°C for 40 min, and then kept for further experiments.

### 2.4 Sensory evaluation

The sensory analysis of treated and untreated AMPS was performed with the evaluation of color, odor, flavor, and overall acceptance. Ten panelists were assigned for the sensory analysis between 25 and 40 years of age, and trained them at least once prior to the experiment. All experimental samples were encoded before sensory evaluation. The panelists scored using a 1–9‐point hedonic scale, where 1 and 9 consisted of an extremely dislike and an extremely like, respectively. (Li, Wang, Fang, & Li, 2013).

### 2.5 Weight loss

Determination of weight loss (%) of AMPS was calculated by the differences in weight before and after oven drying as followed by the method of Goulas and Kontominas (2005).

### 2.6 Total bacterial count (TBC)

The AMPS (1 g) was mixed with 9 ml of sterile saline and then homogenized in sterilized plastic bags for 3 min using a Stomacher 400 Circulator (Seward Limited, West Sussex, UK). Three serial dilutions of the homogenate were made, spread onto plate count agar (Difco, Franklin Lakes, NJ, USA), and incubated at 37°C for 48 hr, as followed by Chen et al. (2015).

Total coliform count in AMPS was performed using a most probable number method. From the same dilutions as mentioned above, each of the homogenate was inoculated onto EC medium and incubated at 35°C for 24 hr for growth and gas production. If no gas production was found in the fermenter tubes, the result was considered to be negative **(−**).

### 2.7 Odor intensity

The odor intensity of AMPS was measured as followed by the previously described method (Macagnano et al., 2005). Briefly, the sample (5 g) was placed in a 50‐ml conical tube which fits the applied odor concentration meter (XP‐329, New Cosmos Electric Co. Ltd., Osaka, Japan). After closing the lid, the odor intensity was monitored until the higher peak signal appeared and was expressed as an arbitrary unit (Macagnano et al., 2005).

### 2.8 Color evaluation

The color on the surface of AMPS was evaluated using a CM‐700d Konica Minolta (Tokyo, Japan) instrument. Following calibration with white reference, the triplicate readings were taken from each sample with a Hunter system values such as L* (lightness), a* (redness), b* (yellowness) as described in a previous study (Bashir et al., 2018; Chen et al., 2015).

### 2.9 Texture analysis

The AMPS fillets were undertaken for instrumental texture analysis using a Brookfield Texture Analyzer (Massachusetts, USA) operated by a software (Texture PRO CT, Middleboro, USA) in the computer. The sample was compressed by 50% of their sample height using an aluminum cylinder probe with a diameter of 10 mm at 0.5 mm/s cross‐head speed. The deformation state was continued for 60 s and then extruded. The textural analysis was performed at room temperature with triplicate measurements of each groups. During compression and extrusion, a number of attributes were measured, including hardness, cohesiveness, springiness, and chewiness as described previously (Ganesan & Benjakul, 2014).

### 2.10 pH measurement

The AMPS (4 g) was homogenized using a homogenizer (SHG‐15D, SciLab, Seoul, Korea) in 45 ml of distilled water (DW) for 2 min. After centrifugation, the supernatant was collected and filtered through Whatman filter paper (Advantec Toyo Kaisha, Ltd., Tokyo, Japan). The pH of the homogenate was monitored using a pH meter with a glass electrode into the homogenate (OHAUS STARTER 2100, Seoul, Korea).

### 2.11 Volatile basic nitrogen (VBN)

Conway microdiffusion method was used to quantitatively assess the level of VBN produced in AMPS (Oğuzhan Yildiz, 2015). AMPS (5 g) was diluted with 25 ml of DW in a glass beaker and then mixed thoroughly to homogenize. After filtration, the sample solution (1 ml) and potassium carbonate (1 ml) were added to the outer chamber and subsequently 0.1 N HCl (1 ml) was added to the inner chamber of Conway unit. The Conway cell was incubated at 37°C for 90 min followed by the titration with 0.01 N NaOH.

### 2.12 Thiobarbituric acid‐reactive species (TBARS)

For quantitative analysis of TBARS, the sample (5 g) was homogenized in 12.5 ml TBARS solution containing 20 trichloroacetic acid with 2 M phosphoric acid, filtered, and then incubated in a water bath at 95°C for 30 min as followed in the previous report (Oğuzhan Yildiz, 2015). After adjusting the sample to room temperature, 200 μl of each sample including blank group (distilled water) was added to each well of the 96‐well plate and recorded the absorbance value at 530 nm wavelength using a nano SPECTRO star (Newtown, UK).

### 2.13 Proximate composition analysis

The proximate chemical compositional analysis of AMPS, including total contents of calories, sodium, carbohydrate, sugars, crude fat, trans fat, saturated fat, cholesterol, crude protein, potassium, calcium, iron, and vitamin D were performed according to the standard method of AOAC (2000) by Traditional Microorganism Resources Center, Keimyung university, Daegu, Korea.

### 2.14 Gas chromatography/Mass spectrometer (GC/MS) analysis

The combined treatment of superheated steam roasting and the hot smoke of AMPS was subjected to headspace‐GC/MS analysis to evaluate the content of trimethylamine N‐oxide (TMAO). The sample (10 g) was placed in a 50‐ml falcon tube with 10 ml DW and then sonicated for 20 min. The supernatant was filtered after centrifugation at 2200 X g for 10 min, transferred to solid phase microextraction system, and then volatilized into the GC instrument (Agilent 7890B GC) at oven temperature 240°C which increased from 40°C to 210°C following the flow rate of 10°C/min. The carrier gas used was Helium, which was mixed with the gaseous compounds of the sample which underwent separation through a DB‐WAX column (30 m length × 0.25 μm i.d; 0.25‐μm thickness). The identification and quantitation of TMAO were performed in superheated and smoked AMPS as compared with standard compound (Sigma‐Aldrich, St. Louise, MO, USA).

### 2.15 Statistical analysis

All experiments were performed in triplicate measurements (*n* = 3), where data were expressed as mean values ± standard deviation (*SD*). Statistical analyses at the probability level of 95% were considered to be statistically significant and set at *p* < .05 measured by one‐way analysis of variance using an SPSS 10 software (SPSS, Chigaco, IL, USA).

## 3 RESULTS AND DISCUSSIONS

### 3.1 Effect of time‐dependent superheated steam on sensory analysis

**Figure 1:**
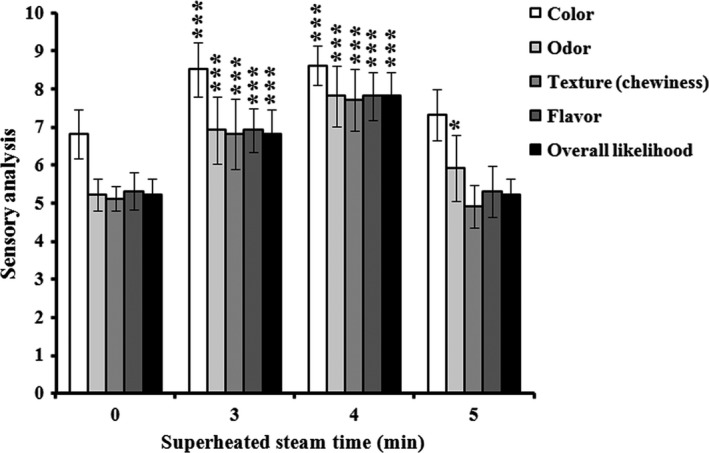
Effect of sensory analyses associated with color, odor, texture (chewiness), flavor, and overall likelihood using superheated steam roasting on AMPS at different heating times (0, 3, 4, and 5 min). Data expressed as mean ± *SD* (*n* = 10). *p < .05 AND ***p < .001 compared with no treatment group (ANOVA)

Currently, superheated steam roasting is a newly introduced, most popular, food processing technique in the food industry and has yielded a nonoxidized food product because of its unique properties, of which, superheated steam is replaced by the air (O_2_) so that the final product can be processed under a nonoxygen condition (Zzaman, Bhat, Yang, & Easa, 2017). It has been reported that superheated steam can act as a drying medium to process foods such as fish meals, shrimps, beet pulp, and sliced raw potatoes, in which the dried foods showed better quality than other conventional drying methods (Blasco & Alvarez, 1999; Iyota, Nishimura, Onuma, & Nomura, 2001; Zzaman et al., 2017). Sensory evaluation based on the criteria for color, odor, texture, flavor, and overall likelihood was scored as good quality by the panelists when AMPS was processed by the superheated steam treatment at running time of 3 and 4 min, respectively (Figure 1). Therefore, we optimized the superheated steam treatment at running time of 4 min for further experiments, because it was rated as highest in sensory evaluation as compared to the running time at 3 min.

### 3.2 Effect of different sawdust smoke on sensory evaluation

**Figure 2:**
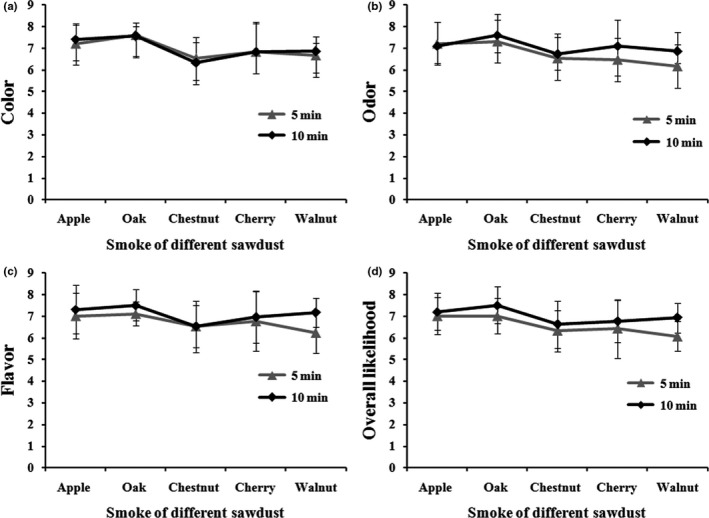
Effect of sensory evaluation associated with color (a), odor (b), flavor (c), and overall likelihood (d) using the smoke treatment of different plant‐derived sawdusts (Apple, Oak, Chestnut, Cherry, and Walnut) on superheated steam roasting of AMPS at two different smoking times (5 and 10 min). Data expressed as mean ± *SD* (*n* = 10)

Studies to improve the sensory quality of fish products have intensively observed with the use of different sawdust materials to smoke (Küçükgülmez, Eslem Kadak, & Celik, 2010), which possess various complex mixtures of volatile chemicals among which phenols are a predominant compound to develop color and aroma in food (Dillon, Patel, & Martin, 1994). For this objective, to develop a color and odor in AMPS after using superheated steam roasting with different sawdust materials, sensory preference criteria such as color, odor, flavor, and overall likelihood were assigned by the panelists. The most liked one was found to be Oak sawdust‐smoked AMPS followed by apple, walnut, cherry, and chestnut sawdusts, at two different smoking times (5 and 10 min) (Figure 2). Therefore, we optimized Oak sawdust as the most preferred smoking materials for further experiments. Similar results have been obtained from previous independent experiments, hot smoking with different sawdust had significant sensorial impacts on smoked Wels catfish (Küçükgülmez et al., 2010).

### 3.3 Effect of smoking time on sensory analysis

**Figure 3:**
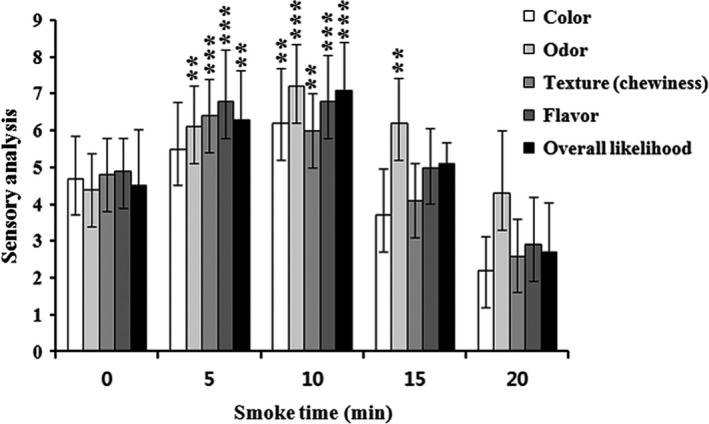
Effect of sensory analyses associated with color, odor, texture (chewiness), flavor, and overall likelihood using an optimized oak sawdust smoke at different smoking times (0, 5, 10, 15, and 20 min) on superheated steam roasting of AMPS. Data expressed as mean ± *SD* (*n* = 10). **p < .01 and ***p < .001 compared with no treatment group (ANOVA)

Incomplete combustion of wood sawdust results in an increase in shelf life with favorable effects on antioxidant and antimicrobial activities have been documented due to various classes of chemicals derived from wood smoke (Dillon et al., 1994). Each chemical contributes to improve the overall sensory characteristics of wood smoke. However, no precise report is available for the prediction of optimal smoking time of AMPS. Superheated steam of AMPS was smoked by oak sawdust time dependently, which resulted in a significant increase in sensory properties scored mostly by the panelists at the smoking time of 10 min followed by 5 min in the present study (Figure 3). Excessive smoking can produce a burning smell incorporated with damaging fish proteins and other essential nutrients, thereby leading to decreased overall sensory quality of smoke.

### 3.4 Effect of smoking time on odor analysis

**Figure 4:**
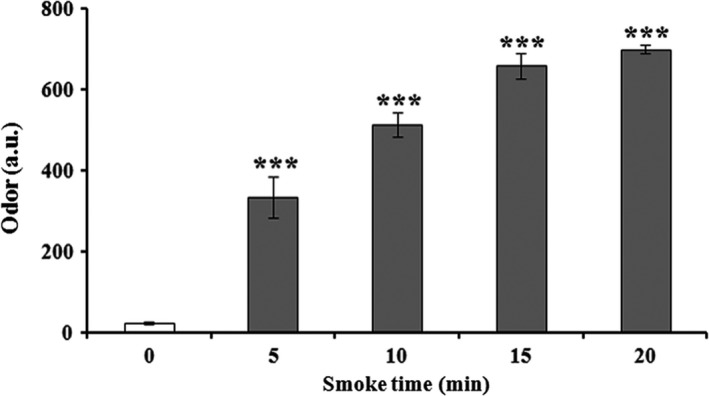
Effect of instrumental odor analysis with the extension of smoking times (0, 5, 10, 15, and 20 min) by oak sawdust on superheated steam roasting of AMPS. Data expressed as mean ± *SD* (*n* = 3). ***p < .001 compared with no treatment group (ANOVA)

To meet the demand of quality measurements in the seafood industry, instrumental odor analysis can be replaced to act as human senses using electronic nose to test odor (Macagnano et al., 2005). Thus, it can improve the performance rather than choosing single sensor technology to assess seafood freshness. The physicochemical interactions between volatile substances and the sensitive films were recorded to a numerical value, indicating the presence of volatile compounds in the processed AMPS. Here, odor intensity significantly increased with the increase in time‐dependent smoking from 0 to 20 min (Figure 4). However, increased levels of odor could have negative impacts in sensory evaluation. Consequentially, we found that a 20‐min smoking time reduced the acceptability of superheated steam AMPS tested by the panelists.

### 3.5 Effect of smoking time on weight loss

**Figure 5:**
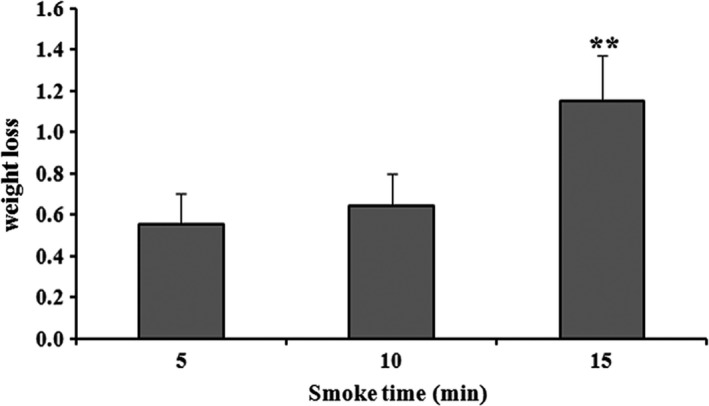
Effect of weight loss with different smoking times (5, 10, and 15 min) by oak sawdust on superheated steam roasting of AMPS. Data expressed as mean ± *SD* (*n* = 3). **p < .01 compared with treatment group at 5 min (ANOVA)

Weight loss in different time‐dependent smoking groups of superheated steam roasting of AMPS revealed no significant difference with 10‐min smoking, but 15‐min smoking significantly increased the weight loss (Figure 5). It was proved that 5‐min and 10‐min smoking time delayed the release of moisture in the processed AMPS, indicating its ability to keep the firmness of the foods during storage, and the results were supported by Oğuzhan Yildiz (2015).

### 3.6 Effect of smoking time on color evaluation

**Figure 6:**
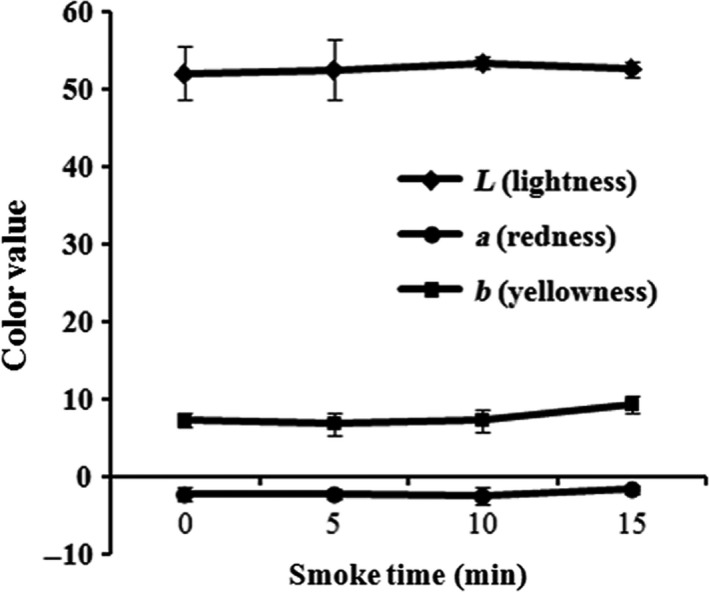
Effect of different smoking times (0, 5, 10, 15, and 20 min) on the color of superheated AMPS. Data expressed as mean ± *SD* (*n* = 3)

Color of foods is sensitive to the consumers while appetite can easily be stimulated with respect to the observer’s reaction to color (Nollet & Toldrá, 2009). The color or lightness of the processed food often indicates the flavor that consumers taste (Downham & Collins, 2000). Time‐dependent smoking of superheated steam AMPS revealed that L (brightness), a (redness), and b (yellowness) were consistent throughout the smoking time (Figure 6). The color attributes were not affected in superheated steam AMPS while treating with hot smoking, and the results were supported by the previous studies (Blasco & Alvarez, 1999; Zzaman et al., 2017).

### 3.7 Effect of smoking time on texture analysis

**Figure 7:**
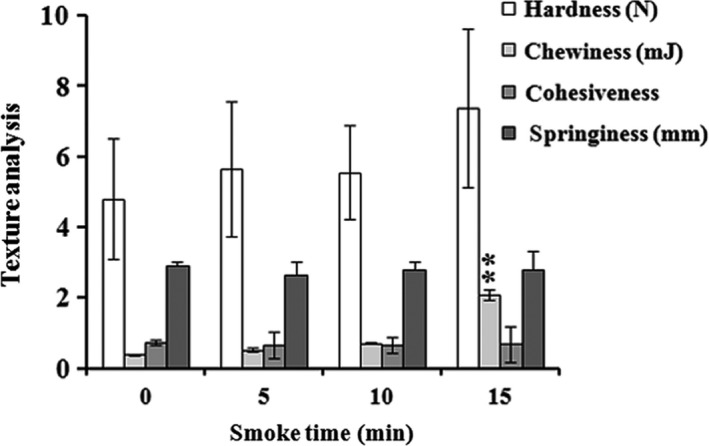
Effect of instrumental texture analyses associated with hardness (N), chewiness (mJ), cohesiveness, and springiness (mm) using an oak sawdust smoke at different smoking times (0, 5, 10, and 15 min) on superheated steam roasting of AMPS. Data expressed as mean ± *SD* (*n* = 3). **p < .01 compared with no treatment group (ANOVA)

Instrumental texture analysis can be applied in the seafood products and measured using several parameters that can easily be achieved during experimentation (Macagnano et al., 2005). To assess the quality features of processed AMPS, the increase in smoking time up to 10 min maintained constant hardness of foods while an increase but nonsignificant hardness was observed at 15‐min smoking time. Cohesiveness and springiness were found to be stable through the smoking time (Figure 7). The changes in chewiness was significant when it was smoked for 15 min. The results suggested that the smoking time had no effect on the texture quality of superheated steam with the hot smoke of AMPS, which might be due to its ability to maintain the air velocity and moisture content of the processed AMPS as evident by Moghaddam, Razavi, Taghizadeh, and Sazgarnia (2016).

### 3.8 Effect of smoking time and storage duration on microbiological changes

**Figure 8:**
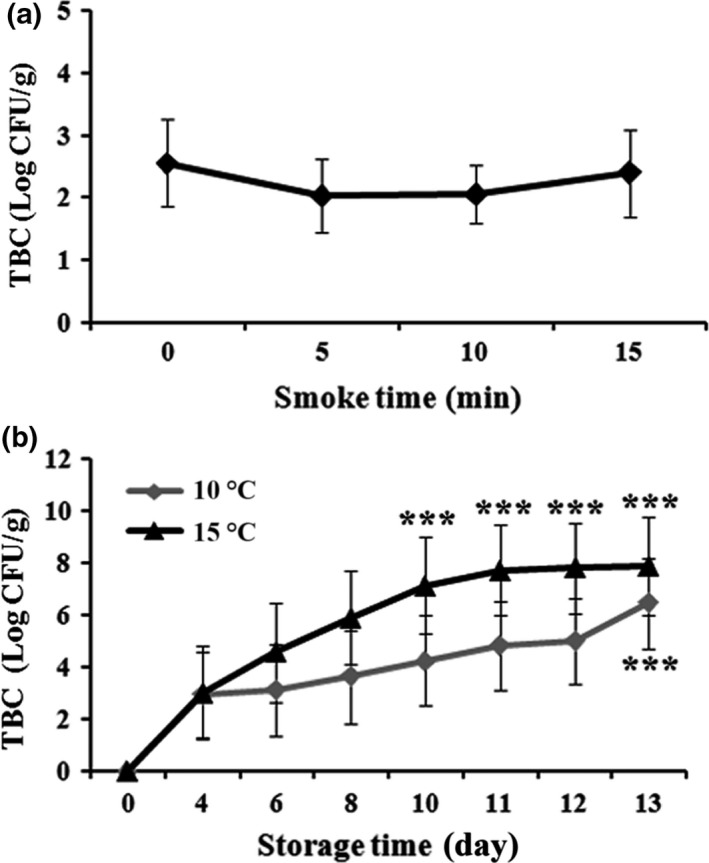
Effect of total bacterial count (Log CFU/g) with different smoking times (0, 5, 10, and 15 min) (a) and storage conditions (10°C and 15°C) from Day 0 to 13 (b) on superheated steam roasting of AMPS. Data expressed as mean ± *SD* (*n* = 3). ***P < 0.001 compared with no treatment group (ANOVA)

Reports from previous studies suggested that superheated steam roasting increased antibacterial effects and extended the shelf life of food products with intact quality (Takashi, 2005). As can be seen in Figure 8a, the hot smoking slightly and non‐significantly decreased the TBC up to the running time at 15 min Therefore, the smoking time was optimized for 10 min in Figure 8b. With an optimal smoking time of 10 min, the storage temperature at 15°C significantly increased TBC during storage at 13 days, while that for 10°C successfully and significantly restricted TBC, especially during at the storage day 4–12. The total coliform count was found to be negative during the entire period of storage (Figure 8b). Chen et al. (2015) reported similar results where superheated steam of AMPS in combination with water‐soluble chitosan prevented bacterial contamination during the frozen storage. However, the smoke of sawdust having plenty of natural volatile compounds with antioxidant properties might add one more attribute here to prevent TBC (Bashir et al., 2017; Goulas & Kontominas, 2005; Guillén & Errecalde, 2002).

### 3.9 Effect of smoking time and storage duration on pH changes

**Figure 9:**
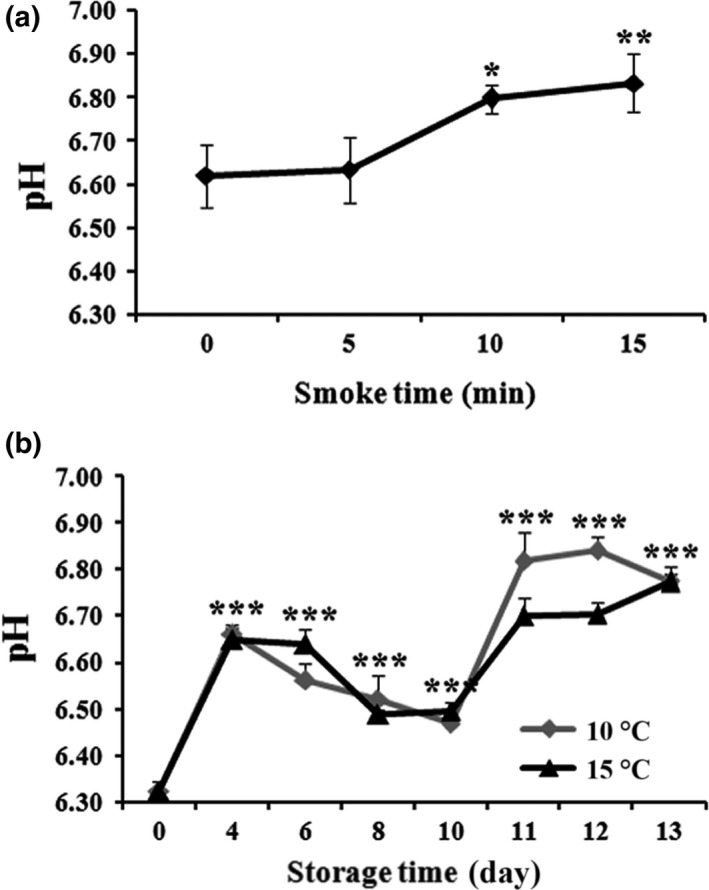
Effect of pH with different smoking times (0, 5, 10, and 15 min) (a) and storage conditions (10°C and 15°C) from Day 0 to 13 (B) on superheated steam roasting of AMPS. Data expressed as mean ± *SD* (*n* = 3). *p < .05, **p < .01, and ***p < .001 compared with no treatment group (ANOVA)

The pH is one of the indicators for assessing fish product quality in the food industry, because it shows depletion in the flesh during storage (Nollet & Toldrá, 2009). Factors such as rigor development, postmortem, and pH often influence the quality in the process technology. The results revealed that the pH measurements were increased up to 6.80 and 6.83 when smoking for 10 and 15 min, respectively, indicating its ability for neutralizing the pH value in the flesh. Moreover, with an optimal smoking time of 10 min, the pH value of superheated steam with the hot smoke of AMPS decreased after 4 days of storage and continued to increase after 12 days to become pH 6.80 as observed in two different storage temperatures at 10°C and 15°C, respectively (Figure 9). The findings were supported by the previous study where AMPS coated with water‐soluble chitosan showed an increased trend of pH from 6.5 to 7.36 during a month‐long frozen storage, which was attributed as basicity of the chitosan (Chen et al., 2015). Moreover, Nollet and Toldrá (2009) demonstrated their report where the postmortem pH of fish varied from 5.5 to 7.1 which depends on season, species, and other factors associated with fish handling and preservation.

### 3.10 Effect of smoking time and storage duration on VBN changes

**Figure 10:**
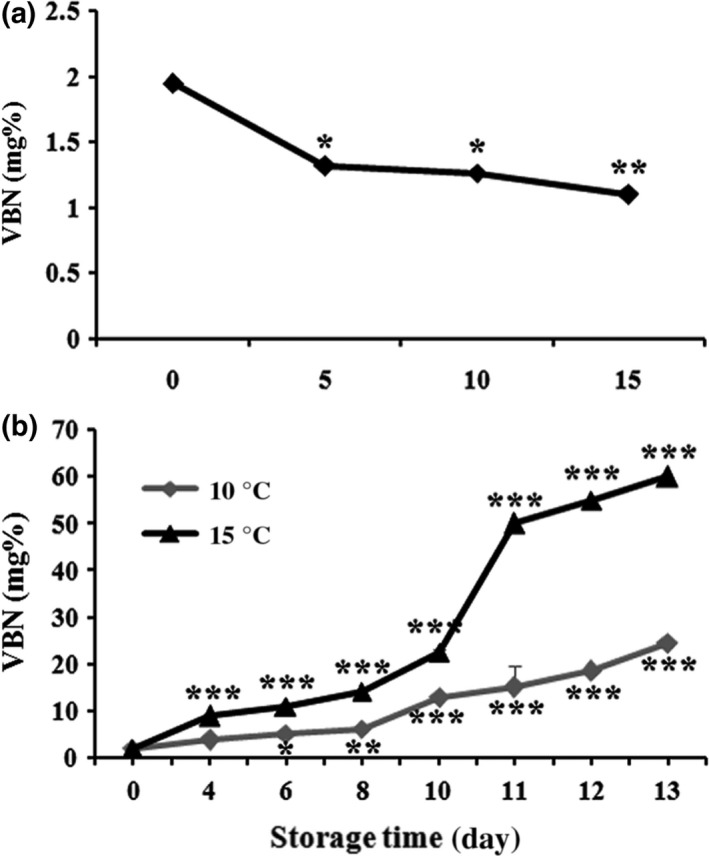
Effect of volatile base nitrogen (VBN mg%) with different smoking times (0, 5, 10, and 15 min) (a) and storage conditions (10°C and 15°C) from Day 0 to 13 (b) on superheated steam roasting of AMPS. Data expressed as mean ± *SD* (*n* = 3). *p < .05, **p < .01, and ***p < .001 compared with no treatment group (ANOVA)

The VBN level is found to be an indicator of measuring the degree of fish and fish products’ spoilage primarily due to ammonia after deamination of amino acids, trimethylamine by spoilage bacteria and autolytic enzymes during frozen storage (Nollet & Toldrá, 2009). In Figure 10a, the VBN value was significantly decreased with the increase in smoking time from 0 to 15 min. At 10 min of smoking time, AMPS was undertaken to measure the VBN at two different storage temperatures (10°C and 15°C) from Day 0 to Day 13. The VBN value was significantly increased from Day 0 (2.12 mg%) to Day 13 (60 mg%) at 15°C storage condition, whereas at 10°C the temperature slowed down the progression of VBN levels from Day 0 (2.12 mg%) to Day 13 (24.43 mg%) (Figure 10b). Comparing these results with literature, the levels of TBN 30–35 mg N/100 g muscle are considerable for consumption in ice‐stored cold‐water fish, whereas the amount of TBN in fresh fish is generally 5–20 mg N/100 g fish muscle (Huss, 1988). Results indicate that superheated steam roasting with the hot smoke of AMPS significantly extended the shelf life at good quality by preventing the TBN production during storage at 10°C.

### 3.11 Effect of smoking time and storage duration on TBARS changes

**Figure 11:**
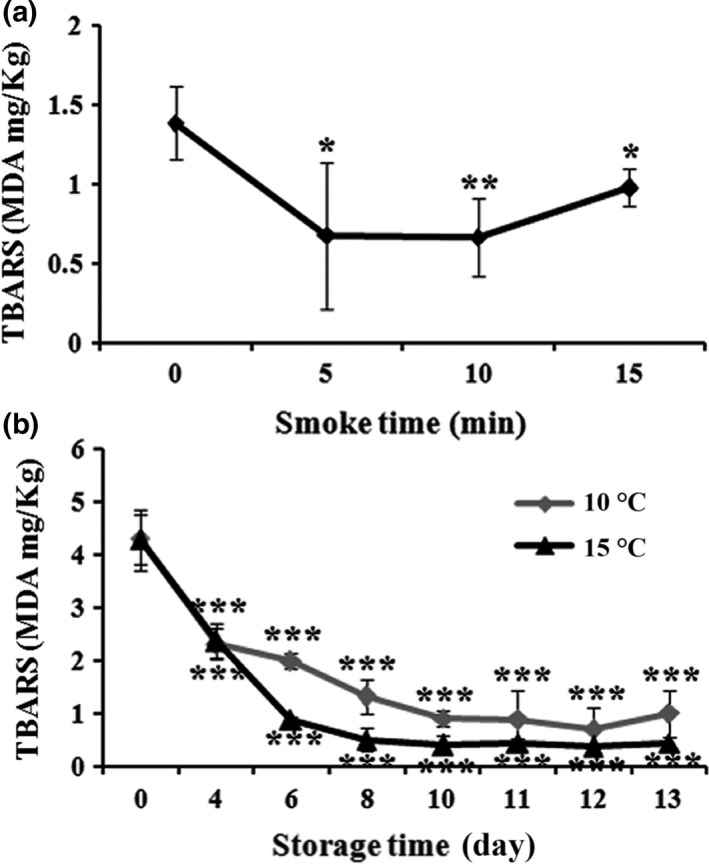
Effect of thiobarbituric acid‐reactive substances (TBARS; MDA mg/kg) with different smoking times (0, 5, 10, and 15 min) (a) and storage conditions (10°C and 15°C) from Day 0 to 13 (b) on superheated steam roasting of AMPS. Data expressed as mean ± *SD* (*n* = 3). *p < .05, **p < .01, and ***p < .001 compared with no treatment group (ANOVA)

Lipid oxidation is the most promising limiting factor in the seafood industry, which results in an off odor and off taste in the final product due to rancidity. Moreover, Nollet and Toldrá (2009) reported that under storage conditions, whether chilled or frozen, lipid oxidation‐derived compounds can destroy the nutritional quality of the fish products by interfering with proteins. The value of TBARS indicates an array of lipid oxidation by measuring the content of malondialdehyde (MDA). MDA is an initial reaction product of polyunsaturated fatty acid oxidation. In Figure 11a, time‐dependent smoking can significantly reduce the content of TBARS in superheated steam roasting AMPS, with an optimal amount of 0.66 MDA mg/kg at the smoking time of 10 min. At this smoking time, processed AMPS gradually decreased the content of MDA from Day 4 to Day 12 and then turned to increase at Day 13 as shown in Figure 11b. Moreover, lipid peroxidation‐limiting effects of AMPS were effective at 15°C storage condition than those observed at 10°C. The consumability range of TBARS is reported between 7 and 8 MDA mg/kg, whereas some others suggested to be not more than 5 MDA mg/kg (Oğuzhan Yildiz, 2015).

### 3.12 Effect of superheated steam roasting with hot smoking of AMPS on nutritional quality assessment

In the aspect of nutritional quality, fish product components may differ upon changing the handling, processing, and storage conditions. In Table 1, the proximate composition such as carbohydrate, protein, and lipid contents of superheated steam AMPS treated with hot smoking were found to be 10.530%, 21.775%, and 0.621% (w/w), respectively, in which trans fats and saturated fats were 0.004% and 0.115% (w/w), respectively; the overall results were in accordance with the previous report (Nollet & Toldrá, 2009). The content of minerals such as sodium, potassium, calcium, and iron were relatively as high as 184.273, 264.489, 9.684, and 0.867 mg/100 g compared with Venugopal (2005) who reported that mineral contents of fish muscle and invertebrates were roughly between 0.6% and 1.5%. wet weight. The amount of calories in the processed AMPS was estimated as 134.809 Kcal/100g higher than the previous study (Venugopal, 2005). To support the present report, these analyses revealed that the processed AMPS might have the ability to possess high nutritional quality.

**Table 1:** Nutritional quality assessment for superheated steam roasting combined with the hot smoke of AMPS

Test items	Unit	Results
Calories	Kcal/100 g	134.809
Sodium	mg/100 g	184.273
Carbohydrate	g/100 g	10.530
Sugars	g/100 g	0.112
Crude fat	g/100 g	0.621
Trans fat	g/100 g	0.004
Saturated fat	g/100 g	0.115
Cholesterol	mg/100 g	26.035
Crude protein	g/100 g	21.775
Potassium	mg/100 g	264.489
Calcium	mg/100 g	9.684
Iron	mg/100 g	0.867
Vitamin D	mg/100 g	Not detected

### 3.13 Effect of superheated steam roasting with hot smoking of AMPS on TMAO changes

**Figure 12:**
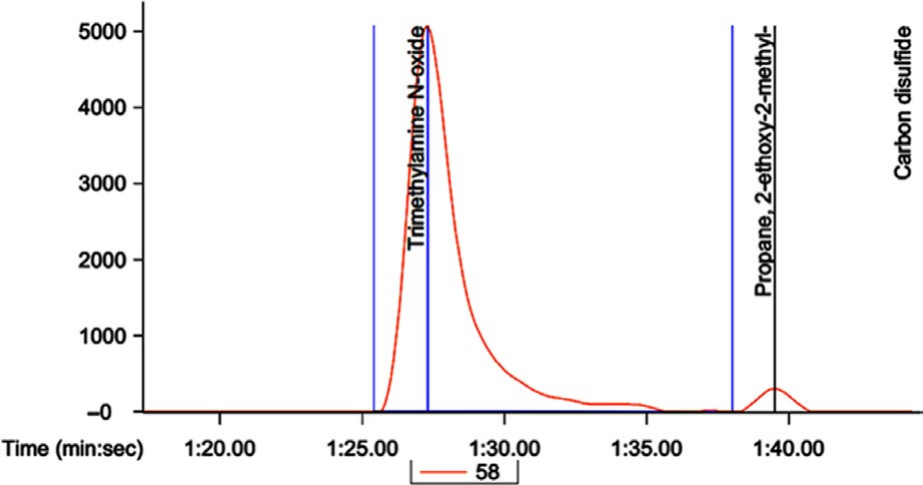
GC‐MS analysis for quantitation of trimethylamine N‐oxide (TMAO) level in processed AMPS

The TMAO level increased with decreasing freshness of fish products that produce an off odor and can be an indicative compound in determining the degree of fish spoilage (Nollet & Toldrá, 2009). Usually, fish often use TMAO as part of their physiological activity such as osmoregulation. However, following the death of fish, TMAO becomes activated to form trimethylamine with the help of bacteria, that is, one of the causes of an off odor (Venugopal, 2005). The TMAO content in seafood appears to be varying degree with respect to their species, age, harvesting time, and environmental factors, and is accounted for 1–100 mg/100 g muscular tissue, while freshwater fish contains 5–20 mg/100 g body weight (Stansby, 1963). In the present study, the processed AMPS showed a low level of TMAO as performed by GC/MS analysis, which was 137 μg/100 g (Figure 12). The result was strongly supported by the previous studies, where TMAO level of AMPS was considered to be safe for human consumption (Nollet & Toldrá, 2009; Stansby, 1963).

## 4 CONCLUSIONS

This study demonstrated the advantages of using superheated steam roasting as an alternative to the conventional heating process, which gave rise to better sensory characteristics of AMPS. Among the different tested sawdust materials to smoke superheated steam roasting AMPS, oak sawdust showed good quality and sensory properties with an optimal smoking time of 10 min. Superheated steam roasting combined with time‐dependent hot smoke showed the desirable odor, negligible weight loss, and constant color and texture properties up to the smoking time of 10 min. At this smoking time, the TBC, pH, VBN, and TBARS values were in suppressive during storage conditions up to 13 days. Moreover, superheated steam roasting in combination with the hot smoke of AMPS achieved better quality food by keeping all essential nutrients at high levels during storage. The processed AMPS contained a very low level of TMAO as compared with that of other seafood products, which were reported to be safe for human consumption. A novel combined method was developed, and thus, it can be utilized to process and preserve AMPS in the food industry. Therefore, using this approach, it is possible to preserve food products, especially the adductor muscle of pen shell, under healthy conditions at the consumer level.

## CONFLICT OF INTEREST

The authors have no competing interests.

## ETHICAL STATEMENTS

Ethical Review: This study was approved by the Institutional Review Board of Animal Care and Use Committee at Silla University (Busan, Korea).

Informed Consent: Written informed consent was obtained from all study participants.
